# Digital Health and Digital Learning Experiences Across Speech-Language Pathology, Phoniatrics, and Otolaryngology: Interdisciplinary Survey Study

**DOI:** 10.2196/30873

**Published:** 2021-11-05

**Authors:** Yuchen Lin, Martin Lemos, Christiane Neuschaefer-Rube

**Affiliations:** 1 Clinic of Phoniatrics, Pedaudiology & Communication Disorders Medical Faculty University Hospital Rheinisch-Westfaelische Technische Hochschule Aachen Aachen Germany; 2 Audiovisual Media Center Medical Faculty University Hospital Rheinisch-Westfaelische Technische Hochschule Aachen Aachen Germany

**Keywords:** digital learning, e-learning, speech-language pathology, phoniatrics, otolaryngology, communication disorders, mobile phone

## Abstract

**Background:**

Advances in digital health and digital learning are transforming the lives of patients, health care providers, and health professional students. In the interdisciplinary field of communication sciences and disorders (CSD), digital uptake and incorporation of digital topics and technologies into clinical training programs has lagged behind other medical fields. There is a need to understand professional and student experiences, opinions, and needs regarding digital health and learning topics so that effective strategies for implementation can be optimized.

**Objective:**

This cross-sectional survey study aims to interdisciplinarily investigate professional and student knowledge, use, attitudes, and preferences toward digital health and learning in the German-speaking population.

**Methods:**

An open-ended, web-based survey was developed and conducted with professionals and students in CSD including phoniatricians and otolaryngologists, speech-language pathologists (German: Logopäd*innen), medical students, and speech-language pathology students. Differences in knowledge, use, attitudes, and preferences across profession, generation, and years of experience were analyzed.

**Results:**

A total of 170 participants completed the survey. Respondents demonstrated greater familiarity with digital learning as opposed to eHealth concepts. Significant differences were noted across profession (*P<*.001), generation (*P=*.001), and years of experience (*P<*.001), which demonstrated that students and younger participants were less familiar with digital health terminology. Professional (*P<*.001) and generational differences were also found (*P=*.04) in knowledge of digital therapy tools, though no significant differences were found for digital learning tools. Participants primarily used computers, tablets, and mobile phones; non–eHealth-specific tools (eg, word processing and videoconferencing applications); and digital formats such as videos, web courses, and apps. Many indicated a desire for more interactive platforms, such as virtual reality. Significant differences were found across generations for positive views toward digitalization (*P<*.001) and across profession for feelings of preparedness (*P=*.04). Interestingly, across profession (*P=*.03), generation (*P=*.006), and years of experience (*P=*.01), students and younger participants demonstrated greater support for medical certification. Commonly reported areas of concern included technical difficulties, quality and validity of digital materials, data privacy, and social presence. Respondents tended to prefer blended learning, a limited to moderate level of interactivity, and time and space–flexible learning environments (63/170, 37.1%), with a notable proportion still preferring traditional time and space–dependent learning (49/170, 28.8%).

**Conclusions:**

This comprehensive investigation into the current state of CSD student and professional opinions and experiences has shown that incorporation of digital topics and skills into academic and professional development curricula will be crucial for ensuring that the field is prepared for the ever-digitalizing health care environment. Deeper empirical investigation into efficacy and acceptance of digital learning and practice strategies and systematic training and practical organizational supports must be planned to ensure adaptive education and practice.

## Introduction

### Background

Rapid technological progress is transforming health care and clinical teaching and learning. Telepractice wearable medical devices, medically certified apps through mobile health (mHealth), health portals, and personalized medicine are among just a few of the many technologies that are increasingly affecting the health care sector. In this context, the multifaceted terms *eHealth* or *digital health*, which encompass many of these technologies, have emerged to describe the evolving means through which technology can be used for information processing and sharing, communication, clinical diagnosis, and treatment to improve human health and well-being [1-4]. Along with these advances in digital health comes the increase in learning through digital means (eg, web courses, simulations, and apps) that allow for learner contact across time and space to promote knowledge creation, expansion, collaboration, and lifelong learning, a phenomenon otherwise known as *e-learning* or *digital learning* [5-8]. The influx of new technological means for health care delivery, teaching, and learning underscores an urgent need to incorporate digital subjects and skills into academic training and professional development milieus [9,10].

However, research has demonstrated that digital skills and the use of digital tools are still not an integral component of professional health care education [11-14]. Suggested explanations for this lack of integration include the demands and competing prioritization of already intensive curricula and the lack of requirements among accrediting bodies [9,15]. Given the current landscape of almost universal ownership of mobile devices among health professionals and students, there is evidence, however, that most health care students prefer web-based resources as their primary source of clinical information [16,17]. Moreover, students often use these devices to access resources for subjects including but not limited to anatomy, drug information, clinical scoring systems, with evidence of increased use of devices during clinical placements [18-20]. However, everyday use of electronic devices does not necessarily translate to effective application in clinical learning contexts. Several studies have shown that students and health professionals are not confident in their eHealth knowledge and skills. In a recent study that surveyed students in 39 European countries, more than half reported poor or very poor eHealth knowledge and skills, and only 40% felt prepared to work in health care contexts increasingly infused with new technologies [21]. Many students cited lack of explicit education on digital skills and tools as a primary reason for feeling unprepared. In the 2016 European Health Parliament survey, more than 80% of the surveyed health care professionals stated that they were insufficiently trained in information and communication technologies and did not feel prepared for developments in eHealth [22]. Considering these findings and rapid technological progress, it is crucial that we begin to examine current student and clinician knowledge, familiarity, and opinions regarding digital health and learning experiences to better incorporate digital skills into clinical education.

In the field of communication sciences and disorders (CSD), investigation into such topics appears to fall behind other medical fields [23,24]. Professionals in CSD treat disorders that affect speech, language, voice, hearing, and the ability to functionally communicate. Although digital technologies such as augmentative and alternative communication (AAC) devices, mathematical-linguistic language modeling, and cochlear implant or hearing aid technologies are well-established [25-27], evidence for digital therapy applications, game-based interventions, and digital screening tools is still emerging [28-33]. Telemedicine and teletherapy are building an evidence base and offer a useful means to deliver services in a patient’s natural environment [34-39]. Despite the influx of new digital solutions, knowledge and implementation of such methods and their quality or efficacy as well as the extent to which these are explicitly incorporated into academic training programs remain unclear [40,41]. For example, while several studies have explored telepractice training for students, formal instruction on such service delivery models appears limited [42-44]; in one study, only 26% of the 97 surveyed universities across the United States indicated formally teaching aspects of telepractice in their programs [44]. As professionals and students in CSD have indicated interest in digital health topics and increased digital learning opportunities, it is essential that digital learning competencies and new therapeutic technologies are deliberately incorporated into clinical education and professional development [40,45,46].

### Objectives

Importantly, CSD is an interdisciplinary field that includes speech-language pathologists (SLPs; German: Logopäd*innen), phoniatricians, and otolaryngologists among other professionals who work closely together to comprehensively treat communication disorders. Given that interdisciplinary education has been identified as a key component to *future proofing* the ever-digitalizing health care environment, it will be useful to investigate interprofessional perspectives and experiences [47-51]. To date, an analysis examining digital health and learning across the interdisciplinary professions involved in CSD has not been conducted, and much existing research primarily comes from the English-speaking population. Examining across profession could be critical to identifying gaps in strategy or implementation and could help to encourage exchange to find collaborative solutions. Thus, this study aims to investigate knowledge, use, attitudes, and preferences toward digital health and learning of current students and professionals across the interdisciplinary fields of speech-language pathology, phoniatrics, and otolaryngology, specifically in the context of German-speaking countries (Germany, Austria, and Switzerland). The potential impacts of profession, generation, and years of experience were also explored.

## Methods

### Overview

The following cross-sectional survey study was conducted in accordance with the guidelines of the Checklist for Reporting Results of Internet E-Surveys (CHERRIES) [52]. In a second part of the survey, the feasibility, attitudes, and preferences for a hypothetical digital learning library app were explored; the results for this second section are presented in a separate article to allow for greater depth of analysis.

### Participants and Recruitment

The study was approved by the Ethics Committee of the Medical Faculty, RWTH Aachen University, and participation was voluntary, anonymous, and could be ended at any time. A convenience sample was collected through sharing an invitational letter and flyer containing a link to the open survey with professional regulating bodies and university clinical programs in speech-language pathology, phoniatrics, and otolaryngology, as well as open student and professional groups on Facebook. To partake in the survey, participants had to be one of the following: (1) physician in phoniatrics or otolaryngology, (2) SLP, (3) medical student, (4) speech-language pathology student (SLP student). Before beginning the survey, participants were prompted to read through the detailed study background, aims, procedures, anonymous data to be collected, and data protection policies; were given the information of relevant contact persons for the study such as the study organizer and data privacy office; and were required to give informed consent before proceeding. No personal information was collected other than demographic information including profession, years of experience, generation, and gender. No incentives were offered.

### Platform

The web-based survey was hosted on university licensed LimeSurvey version 4.3.14+200826, a web-based statistical survey web application that conforms with the required data security legislation as dictated by the German Federal Data Protection Act, the European Data Protection Directive 95/46/EC, and the European General Data Protection Regulation (GDPR) [53]. Unique survey visitors were tracked by cookies as allowed per participant browser settings to prevent repeated access to the survey, though no IP addresses or personal data were saved. Cookies were set at the start of the survey and were valid for the LimeSurvey default of 365 days.

### Survey Design and Content

A semistructured anonymous questionnaire was designed, pretested, and cross-checked by an interdisciplinary team consisting of an SLP, a phoniatrician, and an instructional designer to ensure that questions were clearly formulated and targeted desired data. The survey contained 24 questions pertaining to knowledge, use, attitudes, and preferences regarding digital learning and health as well as sociodemographic information. In total, 1 to 4 questions were displayed per page depending on the question type. There were 12 screens, including the initial page with participant information on which the participant had to give consent before proceeding. The following question types were included in the survey: multiple choice with single fixed choice, multiple answers (with a free-text response option), arrays with Likert scale ratings, yes or no questions, and free-text entries. Directions were provided for each question to avoid confusion (eg, *please choose one of the following answers, multiple answers may be chosen,* and *please rate the following statements*), and technical terms were defined as appropriate to ensure common understanding of the topic or intention of the question. Array questions contained 2 to 5 interrelated statements, which participants rated on a 4-point Likert scale (*translated from German:* disagree, somewhat disagree, somewhat agree, agree). An even-numbered scale was used to avoid central tendency bias, and positive versus negative statements were counterbalanced. Free-text entries were conditionally displayed and followed branching logic based on the preceding yes or no question; they allowed for expansion upon the chosen answer and additional comments. All questions except for free-text entries were mandatory for survey completion and submission. As previous literature has demonstrated no differences in missing data, internal consistency reliability, and mean scale scores across survey participant groups given and not given the option of forward and backward navigation [54], these buttons were included as a safeguard to allow for revision in cases of incorrect responses and to allow for reference to previously defined terms. Responses were collected from August to December 2020. Screenshots of the survey are included in Multimedia Appendix 1.

### Statistical Analysis

Data from the anonymous surveys were analyzed using the IBM SPSS version 27 [55] to generate descriptive summaries of quantitative data. To investigate the potential effects of (1) profession, (2) generation, and (3) years of experience, Kruskal-Wallis H-tests were performed using an α level of *P*<.05 for survey responses involving ranks, such as increasing levels of agreement, familiarity, and frequency (questions 5, 6, 12, 15, 17, 18, 20, and 22). A Bonferroni adjustment was applied for post hoc pairwise comparisons for all reported significant findings. Chi-square tests were implemented using an alpha level of *P*<.05 to determine whether there were associations among (1) profession, (2) generation, and (3) years of experience and knowledge of digital tools, associated concerns, and perceived benefits in terms of time and space (questions 11, 13, 16, 19, and 21). Cramer *V* coefficients provided insight into the strength of associations. Post hoc *Z* tests for significant chi-square tests were also performed.

## Results

### Overview

This study analyzed student and clinician knowledge, use, attitudes, and preferences regarding digital health and learning in CSD in German-speaking countries. Of the 213 unique survey visitors, 13 visited the start page (page containing study information and informed consent) but never began the survey and 29 began the survey though did not complete it. Thus, the participation rate was 93.9% (200/213) and the completion rate was 80.3% (171/213). Only completed questionnaires (optional responses not required) were analyzed. One survey from a student in dentistry could not be used, and thus 170 total surveys were analyzed for the study. Participant characteristics are summarized in Table 1. Generations were defined according to divisions specified by the Pew Research Center [56].

**Table 1:** Participant characteristics.

Characteristics			Values, n (%)
**Gender**			
	Female	150 (88.2)	
	Male	20 (11.8)	
**Profession**			
	Physician (phoniatrician, ENT^a^)	34 (20)	
	Speech-language pathologist	72 (42.4)	
	Medical student (German: *Humanmedizin*)	21 (12.4)	
	Speech-language pathology student	43 (25.3)	
**Generation**			
	Generation Z (1996+)	57 (33.5)	
	Generation Y, millennial (1980-1995)	64 (37.6)	
	Generation X (1965-1979)	35 (20.6)	
	Baby boomer (1946-1964)	14 (8.2)	
**Years of experience**			
	0^b^	61 (35.9)	
	1-5	40 (23.5)	
	6-10	15 (8.8)	
	11-15	11 (6.5)	
	16-20	19 (11.2)	
	>20	24 (14.1)	

^a^ENT: ear, nose, and throat.

^b^Still studying.

### Knowledge

Regarding knowledge of the terms *digital health* and *eHealth*, of the total 170, 20 (11.8%) respondents had heard of the terms and felt confident in their knowledge, 88 (51.8%) respondents indicated having heard of the terms but having limited knowledge, 38 (22.3%) indicated having heard of the terms but being unsure of their meaning, and 24 (14.1%) indicated having never heard of the terms. Kruskal-Wallis tests further revealed significant effects of profession (*H_3_*=30.918; *P<*.001), generation (*H_3_*=15.914; *P=*.001), and years of professional experience (*H_3_*=27.054; *P<*.001) on the level of familiarity with these terms. Pairwise comparisons using the Bonferroni correction revealed that differences were particularly prominent between SLPs and SLP students (*P=*.013), physicians and SLP students (*P<*.001), physicians and medical students (*P<*.001), and physicians and SLPs (*P=*.03), with the former reporting greater familiarity with the terms than the latter. Pairwise comparisons also revealed that Generation X reported greater familiarity with the terms than generations Z (*P=*.002) and Y (*P=*.04). Respondents who were still studying (no experience) tended to be significantly less familiar with the terms *digital health* and *eHealth* than those who had 16 to 20 years of experience (*P=*.001) and those who had more than 20 years of experience (*P=*.005).

In contrast, when respondents were asked about their familiarity with the terms *digital learning* or *e-learning*, 40.6% (69/170) respondents reporting being familiar with and feeling confident with the terms, 54.7% (93/170) indicated being familiar with the terms but having limited knowledge, and 4.7% (8/170) reported having heard of the terms but being unsure of their meaning. No significant differences were found across profession, generation, or years of experience. These terms were explicitly defined following these questions to ensure common understanding for the remainder of the survey. The terms *digital health*, and *eHealth*, were defined as *the use of technology or digital media to promote, maintain, or manage a person's health*. The terms *digital learning* and *e-learning* were defined as “all forms of learning in which electronic or digital media are used to support learning processes, in this case especially in the context of medical or clinical education.”

To similarly establish common understanding, the term *digital therapy tool* was broadly defined as “any electronic or digital media to be used for clinical or clinical research purposes (eg, health tracking, diagnostic tool, therapy exercises).” Regarding familiarity with such tools, 61.8% (105/170) respondents indicated being familiar with such tools, whereas 38.2% (65/170) were not. Significant differences in knowledge of digital therapy tools were found across profession (χ^²^_3_=20.3; *P<*.001), with a moderate effect size (Cramer *V*=0.346; *P<*.001). Post hoc Z tests demonstrated significant differences at the *P=*.05 level for physicians and medical students who were less familiar with digital therapy tools and for SLPs who were more familiar with digital therapy tools. Significant differences were also found across generations (*χ*^²^_3_=8.5; *P=*.04) with a moderate effect size (Cramer *V*=0.224; *P=*.04). Generation Y (millennials) had more respondents who were familiar with digital therapy tools (*P=*.05). No significant differences were demonstrated across years of experience.

A total of 50% (85/170) participants responded to the optional follow-up question “What digital therapy tools are you already familiar with? What did you think of these tools?” Free-text responses consisted of references to general digital therapy tools (19/85, 22.4%; eg, *apps*, *computer programs*, *teletherapy platforms,* and *AAC devices*), specific tools (63/85, 74.1%; eg, *Neolexon, Lexico,* and *Metatalk*), and responses that included both general and specific tools (3/85, 3.5%). Notably, there were several digital health tools mentioned that were unrelated to communication disorders or used for personal use (eg, fitness apps or health insurance apps; n=16). The relevant digital therapy tools that were mentioned included therapy apps (n=77), AAC devices/software (n=13), computer learning software for children (n=19), teletherapy videoconferencing platforms (n=8), computer-based web programs (n=8), diagnostic apps or computer software (n=3), and other (n=4). The most frequently reported digital tools were the therapy apps Neolexon (n=26), Speech Care (n=11), Phonolo (n=6), and Lexico (n=5). Opinions regarding digital therapy tools were mixed. Positive reports included ease of access, increased practice opportunities for patients, user-friendliness of some applications, usefulness of complexity and stimuli settings, and the potential for increasing patient motivation through interactive activities. Reported negative opinions included associated costs, issues with navigation and user-friendliness (especially for older patients), concerns of screen time and distractibility for children, and an emphasis on digital tools only as a supplement to traditional therapy. In relation to associated costs, one respondent added that paid apps often provided greater user support and features.

To ensure common understanding, the term *digital learning tool* was predefined *as* “electronic or digital media that can be used for learning purposes at the undergraduate, graduate, or professional development levels.” In terms of knowledge of digital learning tools, of the total 170, 131 (77.1%) respondents indicated that they were already familiar, whereas 39 (22.9%) were not. No significant differences were found across profession, generation, or years of experience. A total of 62.4% (106/170) participants responded to the optional follow-up question “What digital learning tools are you already familiar with for academic studies, teaching, or continuing education? What did you think of these tools?” Responses consisted of references to general digital learning tools (41/106, 38.7%; eg, *podcasts, webinars,* or *platforms*), specific tools (52/106, 49.1%; eg, *Moodle, KenHub,* or *Zoom*), and responses that included both general and specific tools (13/106, 12.3%). Digital learning tools fell into the general categories of learning management systems (n=50), videoconferencing systems (n=34), web courses or webinars (n=28), videos (n=26), web journals (n=18), apps (n=17), websites and search engines (n=17), learning platforms (n=15), podcasts (n=8), digital notecards (n=7), collaboration platforms (n=7), web polling (n=6), repositories of shared materials (n=5), forums (n=2), e-books (n=2), presentation software (n=3), clinical learning tools (n=2), and other (n=5). In terms of specific resources, the learning management system Moodle (n=33), the videoconferencing platforms Zoom (n=19) and Microsoft Teams (n=9), the video platform YouTube (n=9), and the web journal platform PubMed (n=5) were the most frequently mentioned. Notably, many of the mentioned digital learning tools were not specific to the field of CSD, but rather general to educational milieus. Opinions regarding digital learning tools were mixed. Reported benefits included flexibility and accessibility, usefulness for learning theoretical material, the variety of tools available, and multifunctionalities. In addition, 3 participants mentioned that experiences were often dependent on specific implementation by instructors. Frequently reported concerns included limited direct interaction and opportunity to ask follow-up questions, lack of user-friendliness, the learning curve to use digital tools, and concerns regarding the effectiveness for complex topics or practical skills.

### Use

Digital tool use was primarily descriptively analyzed across devices, software or digital system, formats, and frequency of use. Whereas *devices* referred to specific electronic equipment (eg, computer, smartphone), *software or digital system* referred to commonly used clinical and non– clinical-specific tools or programs serving a specific purpose (eg, word processing software or e-billing), and formats referred to the text-, visual-, or multimedia-based presentation of the information (eg, video or podcast). Students and professionals in CSD appear to use computers (n=168), smartphones (n=137), and tablets (n=90) as their primary devices for digital therapy, teaching, and learning purposes. They also use e-Readers (n=7) and MP3 players (n=6). Other devices mentioned in the optional free response *other* category included *electronic communication supports* (n*=*1), *laptops* (n=2), and *audio recorders* (n=2). Only one respondent used game consoles or virtual reality (VR) equipment for therapy, teaching, or learning purposes. A summary of device use across profession, generation, and years of experience is shown in Table 2.

Word processing software (n=169), presentation software (n=141), videoconferencing software (n=141), research databases (n=115), and spreadsheet software (n=114) were the most frequently reported software or digital systems used among respondents. Respondents also reported using online appointment systems (n=70), electronic health records (n=60), statistics software (n=52), hospital information systems (n=38), e-billing ((n=28), hospital communication systems (n=18), e-prescriptions (n=15), and telemedicine (n=13). In the optional free response *other* category, respondents also reported using *WhatsApp* (n*=*1), *cloud learning platform* (n*=*1), *test evaluation software* (n*=*1), *therapy apps* (n*=*1), *therapy material database* (n*=*1), and *handwritten digital notes via Notability* (n*=*1). A summary of software or digital system used across profession, generation, and years of experience is shown in Table 3.

**Table 2:** Device use across profession, generation, and years of experience.

			Computer		Smartphone		Tablet		e-Reader		MP3		Virtual reality equipment		Game console		Other	
**Profession (number of participants), n (%)**																		
	Physicians	34 (100)		20 (59)		16 (47)		2 (6)		2 (6)		0 (0)		0 (0)		0 (0)		
	SLPs^a^	71 (99)		58 (81)		40 (56)		3 (42)		4 (5)		1 (1)		0 (0)		2 (3)		
	SLP students	43 (100)		38 (88)		17 (40)		1 (2)		0 (0)		0 (0)		0 (0)		3 (7)		
	Medical students	20 (95)		21 (100)		17 (81)		1 (5)		0 (0)		0 (0)		0 (0)		0 (0)		
**Generation (number of participants), n (%)**																		
	Generation Z	56 (98)		53 (93)		31 (54)		3 (5)		0 (0)		0 (0)		0 (0)		0 (0)		
	Generation Y	64 (100)		55 (86)		30 (47)		0 (0)		3 (5)		0 (0)		0 (0)		4 (6)		
	Generation X	34 (97)		23 (66)		23 (66)		3 (9)		3 (9)		0 (0)		0 (0)		1 (3)		
	Baby boomer	14 (100)		6 (43)		6 (43)		1 (7)		0 (0)		1 (7)		0 (0)		0 (0)		
**Experience (years), n (%)**																		
	0^b^	60 (98)		55 (90)		32 (52)		2 (3)		0 (0)		0 (0)		0 (0)		2 (3)		
	1-5	40 (100)		37 (93)		19 (48)		2 (5)		2 (5)		0 (0)		0 (0)		1 (3)		
	6-10	15 (100)		12 (80)		9 (60)		0 (0)		2 (13)		0 (0)		0 (0)		1 (7)		
	11-15	11 (100)		10 (91)		6 (55)		0 (0)		0 (0)		0 (0)		0 (0)		0 (0)		
	16-20	18 (95)		10 (53)		13 (68)		1 (5)		2 (11)		0 (0)		0 (0)		1 (5)		
	>20	24 (100)		13 (54)		11 (46)		2 (8)		0 (0)		1 (4)		0 (0)		0 (0)		

^a^SLPs: speech-language pathologists.

^b^Still studying.

**Table 3:** Software or digital system use across profession, generation, and years of experience.

		Word processing	Videoconferencing	Presentation software	Research databases	Spreadsheet software	Web Appt. system^a^	Electronic health record	Statistics software	Hospital information system	e-Billing	Hospital communication system	e-Prescriptions	Tele- practice	Other	
**Profession (number of participants), n (%)**																
	Physicians	33 (97)	27 (79)	27 (79)	27 (79)	28 (82)	19 (56)	24 (71)	15 (44)	19 (56)	14 (41)	13 (38)	8 (24)	7 (21)	2 (6)	
	SLPs^b^	72 (100)	55 (76)	59 (82)	49 (68)	56 (78)	34 (47)	30 (42)	26 (36)	15 (21)	13 (18)	4 (6)	4 (6)	6 (8)	3 (4)	
	SLP students	43 (100)	42 (98)	42 (98)	30 (70)	22 (51)	10 (23)	3 (7)	8 (19)	0 (0)	1 (2)	1 (2)	3 (7)	0 (0)	0 (0)	
	Medical students	21 (100)	17 (81)	13 (62)	9 (43)	8 (38)	7 (33)	3 (14)	3 (14)	4 (19)	0 (0)	0 (0)	0 (0)	0 (0)	1 (5)	
**Generation (number of participants), n (%)**																
	Generation Z	57 (100)	53 (93)	49 (86)	38 (67)	30 (53)	14 (26)	10 (18)	12 (21)	4 (7)	4 (7)	0 (0)	1 (2)	4 (7)	1 (2)	
	Generation Y	64 (100)	48 (75)	52 (81)	44 (69)	45 (70)	29 (45)	25 (39)	25 (39)	16 (25)	6 (9)	8 (13)	7 (11)	2 (3)	3 (5)	
	Generation X	35 (100)	28 (80)	29 (83)	22 (63)	29 (83)	22 (63)	18 (51)	9 (26)	12 (34)	16 (46)	6 (17)	6 (17)	7 (20)	1 (3)	
	Baby boomer	13 (93)	12 (86)	11 (79)	11 (79)	10 (71)	5 (36)	7 (50)	6 (43)	6 (43)	2 (14)	4 (29)	1 (7)	0 (0)	1 (7)	
**Experience (years), n (%)**																
	0^c^	61 (100)	56 (92)	53 (87)	38 (62)	29 (48)	17 (28)	7 (11)	10 (16)	4 (7)	2 (3)	0 (0)	3 (5)	0 (0)	1 (2)	
	1-5	40 (100)	29 (73)	31 (78)	29 (73)	28 (70)	13 (33)	13 (33)	17 (43)	5 (13)	7 (18)	3 (8)	3 (8)	5 (13)	3 (8)	
	6-10	15 (100)	12 (80)	12 (80)	9 (60)	12 (80)	9 (60)	10 (67)	6 (40)	8 (53)	2 (13)	3 (20)	2 (13)	1 (7)	0 (0)	
	11-15	11 (100)	9 (82)	10 (91)	9 (82)	11 (100)	8 (73)	8 (73)	6 (55)	5 (45)	1 (9)	3 (27)	0 (0)	3 (27)	1 (9)	
	16-20	18 (95)	15 (79)	13 (68)	12 (63)	15 (79)	8 (42)	9 (47)	6 (32)	5 (26)	8 (42)	4 (21)	3 (16)	4 (21)	1 (5)	
	>20	24 (100)	20 (83)	22 (92)	18 (75)	19 (79)	15 (63)	13 (54)	7 (29)	11 (46)	8 (33)	5 (21)	4 (17)	0 (0)	0 (0)	

^a^Web Appt. system: Web Appointment system.

^b^SLPs: speech-language pathologists.

^c^Still studying.

**Figure 1:**
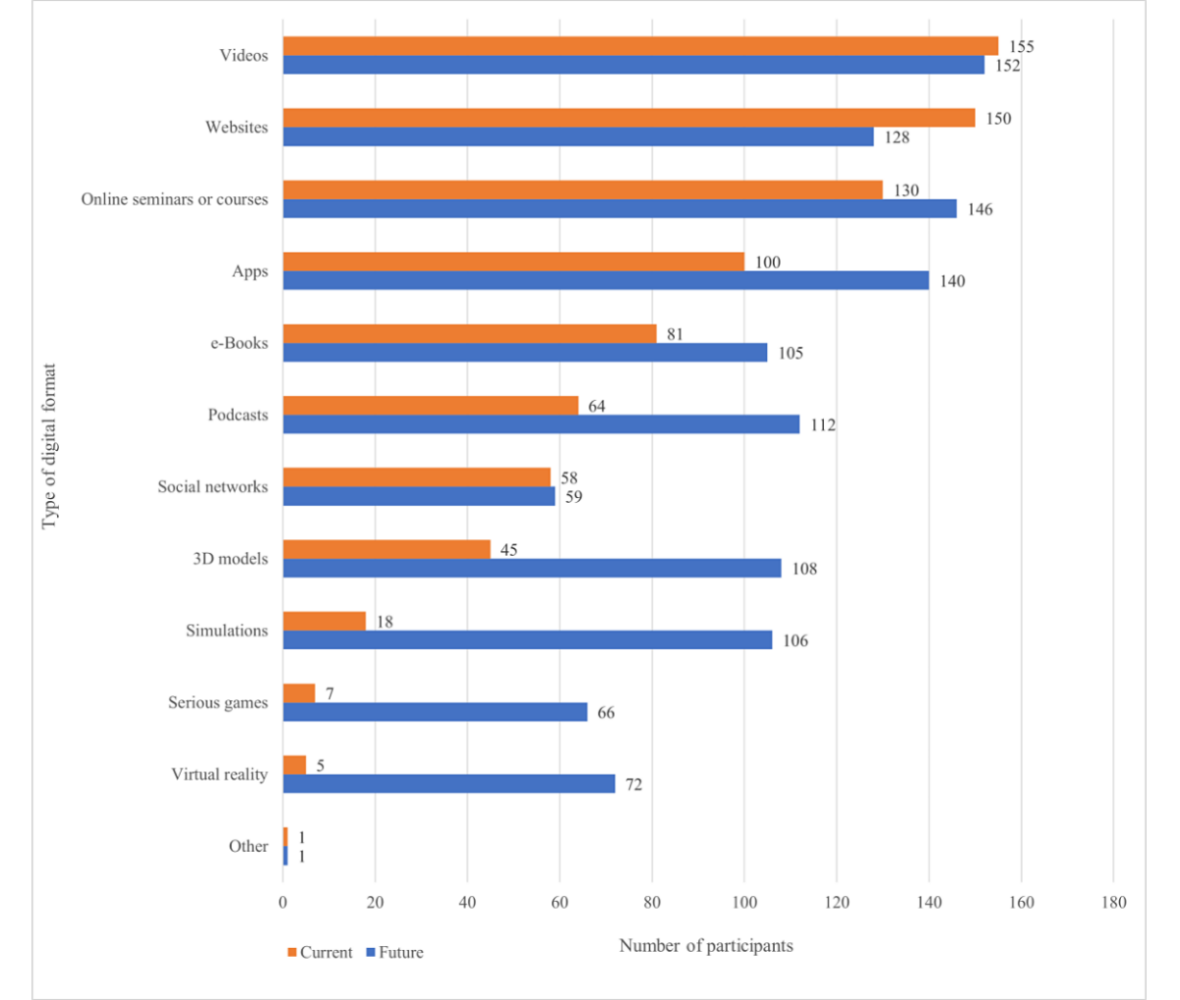
Current and future use of digital format among study participants.

In terms of digital formats, respondents most frequently used videos (n=155), websites (n=150), web-based seminars or courses (n=130), and apps (n=100). When compared with digital formats that respondents could imagine themselves using in the future, many respondents reported also being open to other currently less frequently available digital formats including podcasts (n=112), 3D models (n=108), simulations (n=106), e-books (n=105), VR (n=72), serious games (n=66), and social networking (n=59). In the *other* category, respondents mentioned *learning platforms such as Moodle and Ilias* (n*=*1) and the Promethean ActivTable for Education. These results are shown in Figure 1. A summary of current and future digital format use across profession, generation, and years of experience is shown in Table 4.

**Table 4:** Current (C) and future (F) digital format use across profession, generation, and years of experience.

		Videos	Websites	Web seminars or courses	Apps	e-Books	Podcasts	Social networks	3D models	Simula- tions	Serious games	Virtual reality	Other
**Profession (number of participants), n (%)**													
	Physicians (C)	31 (91)	29 (85)	28 (82)	11 (32)	13 (38)	13 (38)	5 (15)	8 (24)	6 (18)	1 (3)	3 (9)	0 (0)
	Physicians (F)	30 (88)	27 (79)	28 (82)	24 (71)	18 (53)	22 (65)	6 (18)	21 (62)	19 (56)	4 (12)	14 (41)	0 (0)
	SLPs^a^ (C)	64 (89)	65 (90)	57 (79)	44 (61)	33 (46)	28 (39)	33 (46)	16 (22)	3 (4)	2 (3)	1 (1)	1 (1)
	SLPs (F)	66 (92)	56 (78)	65 (90)	63 (88)	46 (64)	46 (64)	30 (42)	48 (67)	47 (65)	35 (49)	34 (47)	1 (1)
	SLP Students (C)	41 (95)	37 (86)	33 (77)	27 (63)	22 (51)	10 (23)	9 (21)	14 (33)	5 (11)	1 (2)	0 (0)	0 (0)
	SLP students (F)	37 (86)	29 (67)	37 (86)	33 (77)	26 (60)	28 (65)	13 (30)	24 (56)	23 (53)	19 (44)	12 (28)	0 (0)
	Medical students (C)	19 (90)	19 (90)	12 (57)	18 (86)	13 (62)	13 (62)	11 (52)	7 (33)	4 (19)	3 (14)	1 (5)	0 (0)
	Medical students (F)	19 (90)	16 (76)	16 (76)	20 (95)	15 (71)	16 (76)	10 (48)	15 (71)	17 (81)	8 (38)	12 (57)	0 (0)
**Generation (number of participants), n (%)**													
	Generation Z (C)	53 (93)	50 (88)	39 (68)	40 (70)	32 (56)	22 (39)	23 (40)	18 (32)	7 (12)	4 (7)	1 (2)	0 (0)
	Generation Z (F)	52 (91)	44 (77)	48 (84)	50 (88)	37 (65)	42 (74)	24 (42)	36 (63)	40 (70)	29 (51)	21 (37)	0 (0)
	Generation Y (C)	58 (90)	55 (86)	50 (78)	41 (64)	26 (41)	18 (28)	26 (41)	15 (23)	4 (6)	2 (3)	0 (0)	1 (2)
	Generation Y (F)	56 (88)	45 (70)	53 (83)	53 (83)	39 (61)	39 (61)	23 (36)	42 (66)	37 (58)	23 (36)	26 (41)	1 (2)
	Generation X (C)	31 (89)	32 (91)	29 (83)	13 (37)	18 (51)	19 (54)	8 (23)	9 (26)	5 (14)	1 (3)	3 (9)	0 (0)
	Generation X (F)	31 (89)	27 (77)	32 (91)	27 (77)	22 (63)	23 (66)	9 (26)	20 (57)	21 (60)	11 (31)	17 (49)	0 (0)
	Baby boomer (C)	13 (93)	13 (93)	12 (86)	6 (43)	5 (36)	5 (36)	1 (7)	3 (21)	2 (14)	0 (0)	1 (7)	0 (0)
	Baby boomer (F)	13 (93)	12 (86)	13 (93)	10 (71)	7 (50)	8 (57)	3 (21)	10 (71)	8 (57)	3 (21)	8 (57)	0 (0)
**Experience (years), n (%)**													
	0^b^ (C)	58 (95)	53 (87)	42 (69)	44 (72)	34 (56)	22 (36)	19 (31)	19 (31)	9 (15)	4 (7)	1 (2)	1 (2)
	0 (F)	54 (89)	43 (70)	51 (84)	51 (84)	39 (64)	42 (69)	22 (36)	38 (62)	39 (64)	28 (46)	23 (38)	0 (0)
	1-5 (C)	35 (88)	35 (88)	31 (78)	25 (63)	21 (53)	11 (28)	22 (55)	10 (25)	1 (3)	1 (3)	0 (0)	0 (0)
	1-5 (F)	35 (88)	30 (75)	33 (83)	33 (83)	26 (65)	23 (58)	15 (38)	27 (68)	26 (65)	16 (40)	15 (38)	0 (0)
	6-10 (C)	13 (87)	14 (93)	12 (80)	9 (60)	4 (27)	6 (40)	6 (40)	4 (27)	0 (0)	0 (0)	0 (0)	0 (0)
	6-10 (F)	15 (100)	12 (80)	14 (93)	14 (93)	8 (53)	10 (67)	7 (47)	9 (60)	6 (40)	6 (40)	4 (27)	0 (0)
	11-15 (C)	11 (100)	8 (73)	10 (91)	5 (45)	3 (27)	6 (55)	4 (36)	2 (18)	1 (9)	1 (9)	1 (9)	0 (0)
	11-15 (F)	10 (91)	9 (82)	8 (73)	9 (82)	6 (55)	9 (82)	6 (55)	8 (73)	9 (82)	5 (45)	8 (73)	1 (9)
	16-20 (C)	16 (84)	17 (89)	16 (84)	7 (37)	7 (37)	9 (47)	2 (11)	5 (26)	2 (11)	1 (5)	0 (0)	0 (0)
	16-20 (F)	15 (79)	14 (74)	16 (84)	17 (89)	10 (53)	14 (74)	2 (11)	12 (63)	11 (58)	6 (32)	8 (42)	0 (0)
	>20 (C)	22 (92)	23 (96)	19 (79)	10 (42)	12 (50)	10 (42)	5 (21)	5 (21)	5 (21)	0 (0)	3 (13)	0 (0)
	>20 (F)	23 (96)	20 (83)	24 (100)	16 (67)	16 (67)	14 (58)	7 (29)	14 (58)	15 (63)	5 (21)	14 (58)	0 (0)

^a^SLPs: speech-language pathologists.

^b^Still studying.

When asked about frequency of digital therapy tool use, of the total 170, 47 (27.6%) respondents indicated that they were still studying, 60 (35.3%) respondents indicated never using digital therapy tools, 27 (15.9%) indicated monthly, 26 (15.3%) indicated weekly, and 10 (5.9%) indicated daily. Significant effects for profession (*H_3_*=15.200; *P=*.002), generation (*H_3_*=12.184; *P=*.007), and years of experience (*H_3_*=20.807, *P=*.001) were found. Post hoc comparisons revealed that SLPs used digital therapy tools significantly more frequently than SLP students (*P=*.01) and medical students (*P=*.03). Across generations, Generation Z reportedly used digital therapy tools significantly less frequently than Generation Y (*P=*.01) and X (*P=*.03), which can likely be attributed to the fact that these individuals are typically still studying and thus have yet to implement these tools in practice. Finally, respondents with 1 to 5 years of experience were noted to use digital therapy tools at a significantly greater frequency than respondents who were still studying (*P=*.001).

Regarding digital learning tools, 18.8% (32/170) respondents never used such tools, 31.2% (53/170) used these monthly, 32.9% (56/170) used these weekly, and 17.1% (29/170) used digital learning tools daily. Significant differences were found across generation (*H_3_*=11.447; *P=*.01) and years of experience (*H_3_*=12.476; *P=*.03). Post hoc comparisons revealed that Generation Z used digital learning tools significantly more frequently than Generation Y. No significant differences were found across years of experience in post hoc analyses.

### Attitudes

Most of the respondents held positive views regarding digitalization in medicine. When asked whether they viewed digitalization positively, of the total 170, 68 (40%) respondents agreed, 90 (52.9%) somewhat agreed, 11 (6.5%) somewhat disagreed, and 1 (0.6%) disagreed. Significant generational differences were found (*H_3_*=18.604; *P<*.001) with Generation Z viewing digitalization significantly more positively than Generation Y (*P=*.003), X (*P=*.02), and baby boomers (*P=*.008). When asked whether they felt prepared for the digital revolution, of the 170, only 29 (17.1%) respondents fully agreed, 78 (45.9%) somewhat agreed, 58 (34.1%) somewhat disagreed, and 5 (2.9%) fully disagreed. Significant differences were found across profession (*H_3_*=8.522; *P=*.04) specifically with SLPs reporting greater preparedness than SLP students (*P=*.04). In total, of the 170, 105 (61.8%) respondents fully agreed and 60 (35.3%) somewhat agreed that eHealth, digital tools, and competences needed to be integrated into future curricula, whereas 5 (2.9%) individuals somewhat disagreed.

Regarding digital therapy tool attitudes, of the 170, 91 (53.5%) respondents agreed, 74 (43.5%) somewhat agreed, and 5 (2.9%) somewhat disagreed to being open to the use of digital therapy tools. In total, 28.2% (48/170) respondents agreed and 41.2% (70/120) somewhat agreed that digital therapy tools offered more advantages than disadvantages with the potential to individualize therapy, whereas 28.8% (49/170) somewhat disagreed and 1.8% (3/170) disagreed. A total of 2.9% (5/170) respondents agreed and 32.4% (55/170) somewhat agreed that they doubted the quality or validity of digital therapy tools, whereas 53.5% (91/170) somewhat disagreed and 11.2% (19/170) fully disagreed. When asked whether they would be more likely to use a digital therapy tool if it were medically certified, of the 170 respondents, 87 (51.2%) respondents agreed, 63 (37.1%) somewhat agreed, 16 (9.4%) somewhat disagreed, and 4 (2.4%) disagreed. Significant differences were demonstrated across profession (*H_3_*=8.806; *P=*.03), generation (*H_3_*=12.499; *P=*.006), and years of experience (*H_3_*=14.270; *P=*.01). Pairwise comparisons revealed no significant differences across professions, though Generation Z tended to agree with using medically certified products more strongly than Generation X (*P=*.008). Across years of experience, respondents who were still studying agreed more with the use of medically certified products than their counterparts with ≥20 years of experience (*P=*.02). When asked whether they would pay a fair price for a digital therapy tool given good ratings, of the 170, 38 (22.4%) respondents agreed, 103 (60.6%) somewhat agreed, 26 (15.3%) somewhat disagreed, and 3 (1.8%) disagreed.

When asked about concerns regarding digital therapy tools, 8.8% (15/170) respondents indicated no concerns, 55.9% (95/170) indicated technical difficulties, 49.4% (84/170) indicated limited validity of digital therapy tools, and 72.9% (124/170) indicated insufficient diagnostic and therapeutic quality of tools. A total of 12.9% (22/170) participants indicated *other* concerns in the free response field. Of these, concerns regarding patient use in terms of patient resistance to such technologies, limited reliability and consistency of use, limits in terms of the individualization of care, potential associated costs, and the potential of cognitive overload especially for older patients were mentioned (n*=*10). In total, 2.9% (5/170) respondents emphasized problems with data privacy and security as an additional concern; 2.4% (4/170) respondents also emphasized the value of direct face-to-face therapy and human interactions, with 0.6% (1/170) respondent specifically citing the negative effects of screen time on child language and social development. Other notable concerns included legal repercussions in cases of medical malpractice and the current lack of sufficient knowledge and exchange regarding digital therapy tools.

Regarding digital learning tool attitudes, of the 170, 125 (73.5%) respondents agreed, 42 (24.7%) somewhat agreed, and 3 (1.8%) somewhat disagreed to being open to the use of digital learning tools. In total, 41.8% (71/170) respondents agreed, 44.7% (76/170) somewhat agreed, 12.4% (21/170) somewhat disagreed, and 1.2% (2/170) disagreed that digital learning offered more advantages than disadvantages. Furthermore, 0.6% (1/170) respondent agreed, 7.1% (12/170) somewhat agreed, 62.9% (107/170) somewhat disagreed, and 29.4% (50/170) disagreed that they doubted the quality and validity of digital learning tools. Many respondents agreed (97/170, 57.1%) and somewhat agreed (57/170, 33.5%) that they would be more likely to use a digital learning tool if it were to be developed by an academic institution or professional regulating body, whereas 5.9% (10/170) somewhat disagreed and 3.5% (6/170) fully disagreed. In total, 21.8% (37/170) respondents agreed, 44.7% (76/170) somewhat agreed, 26.5% (45/170) somewhat disagreed, and 7.1% (12/170) disagreed that they felt confident in their knowledge of digital learning tools.

When asked about their concerns regarding digital learning tools, 11.8% (20/170) respondents reported no concerns, 52.4% (89/170) reported technical difficulties, 45.3% (77/170) reported questionable quality of learning material, 40% (68/170) reported difficulties with self-discipline and sufficient learning competence, and 52.4% (89/170) had concerns regarding reduced social interaction. A total of 10.6% (18/170) participants indicated *other* concerns in the free response field. Of these 18 responses, 2 respondents’ answers expanded upon closed answer choices (eg, *lack of discussion partners* in relation to the answer choice *concerns regarding potentially reduced social interaction*). Five respondents expanded upon concerns regarding the quality of learning, citing the difficulty to verify the quality of digital learning tools, compromised retention of knowledge, the questionable practical relevance of digital learning for the development of interpersonal skills, and fears regarding the potential depreciation of therapeutic competence. Other concerns mentioned included limited scope and specificity of learning materials, difficulty with tracking attendance and engagement, extensive preparation time for digital material, associated costs, and compromised data privacy.

### Preferences

Respondents were asked to indicate their preferred level of virtuality, interactivity, and flexibility in terms of time and space. Out of the 170 total respondents, 4 (2.4%) indicated that they preferred in-person learning, 39 (22.9%) preferred in-person learning with additional e-material, 105 (61.8%) respondents preferred blended learning, and 22 (12.9%) preferred the inverted classroom approach. In terms of interactivity, 21 (12.4%) respondents preferred a passive level, 69 (40.6%) preferred a limited level, 63 (37.1%) preferred a moderate or complex level, and 17 (10.0%) preferred an advanced or active level. No significant differences across profession, generation, or years of experience were found regarding the preferred level of virtuality and interactivity. In terms of time and space flexibility, 28.8% (49/170) respondents preferred traditional time and space–dependent learning environments, 3.5% (6/170) preferred time flexibility only, 30.6% (52/170) preferred space flexibility only, and 37.1% (63/170) respondents preferred time and space–flexible learning environments. As data did not meet chi-square test assumptions for the minimal number of observations per cell, significant findings across profession and years of experience are not reported.

## Discussion

### Principal Findings

This cross-sectional survey study is, to the best of our knowledge, one of the first to investigate knowledge, use, attitudes, and preferences regarding digital health and learning topics interdisciplinarily across the fields of speech-language pathology, phoniatrics, and otolaryngology, which work collaboratively together to treat communication disorders. Although some previous studies have investigated some of these aspects within each respective field, it is useful to analyze across disciplines to identify knowledge gaps, areas for improvement, and collaborative problem-solving opportunities.

### Knowledge

Participants overall reported greater surface level knowledge regarding terminology and specific therapeutic and learning tools. When presented with the terms *digital health* and *eHealth*, only 11.8% (20/170) of respondents indicated feeling confident in their knowledge of the terms. This was especially evident between students and working professionals, the latter of which indicated greater familiarity with terminology as they are more likely to encounter such concepts in their clinical practice. Moreover, the significant finding that older generations and professionals with more experience had greater familiarity of such terminology than their younger and less experienced counterparts highlights the urgency to make eHealth and digital skills an integral part of clinical curricula. In previous studies, health science students similarly reported uncertainty with information technology in health care settings, even if they were confident using technologies in everyday situations. An automatic transfer of digital skills to professional contexts cannot be assumed simply because students are *digital natives* [12,15,57,58]. In fact, the literature has shown that students develop their understandings of eHealth through exposure with concepts and thus require direct instruction and scaffolding. These can in turn help to build confidence with eHealth technologies and contextualized clinical skills [15,40]. Although a 2021 study found that 16 universities across Germany had integrated digital competence–related coursework in their curricula, the extent of integration varied significantly, and many only included elective coursework [59]. Exploring successes and areas for improvement in these examples could serve as a useful starting point from which to explore effective and expanded implementation. Notably, SLPs also demonstrated significantly less familiarity with these terms than their physician counterparts. This finding could potentially be explained by the fact that digital health developments and legislation, such as the Digital Healthcare Act (*Digitale-Versorgung-Gesetz*) in Germany has primarily been medicine-focused and has not equally engaged all clinical fields [60]. Given that quality patient care requires the coordinated efforts of interdisciplinary professionals, it will be crucial that allied medical fields are more deliberately included into digital health developments and legislative action moving forward.

In contrast, respondents reported being more knowledgeable regarding the terms *digital learning* and *e-learning*. This is not surprising given that the term *e-learning* emerged in the 1990s and has since even spread to outside academic contexts, whereas the definition of the term *eHealth* has been debated since the early 2000s, and the debate continues today [61-64]. Although the terms *digital health* and *eHealth* have often been used interchangeably, the World Health Organization has suggested that *digital health* refers to the general use of technologies for health and can encompass eHealth, which refers to the application of health care information technologies (eg, e-billing or e-prescriptions) [65]. However, the fact that over half of respondents still indicated limited knowledge regarding these terms demonstrates the continued need to deliberately familiarize and integrate digital learning tools into learning milieus. This is especially important amidst the ongoing COVID-19 pandemic when much academic learning and professional tasks have shifted onto digital platforms, calling for increased inquiry into effective methods for facilitating an effective and continued digital shift [66]. Learners must be invited to co-design and evaluate digital learning curricula and eHealth technologies so that they feel more prepared to adopt such strategies and adjust as innovations continue to emerge [67].

Most of the respondents indicated knowing digital therapy tools, especially SLPs when compared with physicians and medical students. Although this finding could be explained by different professional scopes of practice, it could also reveal a gap that interdisciplinary collaboration could help to close. Although there is existing literature regarding digital applications in otorhinolaryngology and phoniatrics in the German-speaking population [68,69], these were not well-known among the surveyed physicians and medical students, indicating a need for greater engagement of students and professionals in the evaluation of such tools. Given the surge of eHealth apps during Generation Y’s entrance into the workforce, it is also not surprising that they reported significantly greater knowledge of digital therapy tools, having also been labeled as the most *health-conscious generation* [70-73]. Respondents reported general and specific tools, including digital health tools unrelated to CSD but rather for personal health use (eg, fitness apps). Consistent with previous reports, these findings suggest that student and professional understandings of digital health and therapy tools seem to be informed by personal experiences as health consumers and reflect both evidence- and non–evidence-based tools they may have encountered in academic settings [15,72,74]. Furthermore, positive comments such as increased accessibility, options to adjust complexity, and the potential for increasing patient motivation, as opposed to concerns surrounding costs, user-friendliness, distractibility, and screen time were consistent with previous studies [33,45,75,76].

Most respondents (131/170, 77.1%) reported that they were familiar with digital learning tools. General and specific tools were reported with learning management systems (eg, Moodle), videoconferencing systems (eg, Zoom), and web-based courses or webinars predominating, which may reflect the surge of these platforms during the ongoing COVID-19 pandemic [77]. Few digital learning tools specific to CSD were reported despite their growing number; given that such tools direly require evaluation by current and future professionals, it could be useful in future studies to investigate engagement and familiarization with field-specific learning tools [40,78-82]. Additional comments made by respondents praising the flexibility and accessibility of digital learning tools as well as its drawbacks such as limited interaction and exchange and technical challenges were in line with previous research [83-85]. The mention of instructor-specific implementation affecting the digital learning experience also emphasizes a need for improved systematic training of clinical instructors in effective digital learning implementation [15,21,40].

### Use

In line with commonly reported device use, professionals and students mostly used computers, smartphones, and tablets for learning, teaching, and professional purposes [18,19,83,86]. Game consoles and VR devices were only used by one respondent, despite their growing presence in the literature across the surveyed professions [87-89]. Although an evidence base for such devices is still emerging and ethical considerations must be further evaluated, VR could provide an engaging means through which to deliver both clinical training and therapeutic activities that could be generalized to real-world tasks [89]. In terms of software or digital systems, technologies more closely related to eHealth such as electronic health records, telepractice, and hospital or clinic information or communication systems were mostly used by working professionals, though were still used to a lesser extent than word processing, presentation, spreadsheet, and videoconferencing software. This could reflect the reported lag in eHealth uptake in Germany despite significant progress in legislation for digital health care in recent years [90]. In the Bertelsmann Foundation 2018 international comparison of digital health index scores, Germany still ranked second to last among 17 surveyed Organization for Economic Cooperation and Development countries [86]. Socioculturally, Germany’s lag in digital uptake and focus on data security and privacy have been associated with the country’s problematic and traumatic history of heavy surveillance. This has inevitably affected the country’s now cautious approach to digitalization [91,92]. However, considering reports of increasing acceptance of and demand for eHealth technologies, this trend will likely change in the coming years [91,93]. This can be seen in respondents’ feedback regarding digital formats they could imagine using in the future. Many expressed a desire for learning with and implementing newer, often more interactive formats in the future, such as 3D models, simulations, and serious games. Notably, in the optional free response *other* category across questions pertaining to use of digital technology, respondents often did not differentiate among devices, software or digital systems, and formats, indicating a pressing need for foundational digital literacies education [2,21,22]. Here it is useful to mention that use of devices, software or digital systems, and digital formats were not separately analyzed between digital therapy tools versus digital learning tools as these are common to both types of tools. Nevertheless, a separate analysis in future studies could help with insights into the appropriateness of certain device, software, or format types for specific use cases.

In terms of frequency of use, the largest proportion of respondents (60/170, 35.3%) surprisingly responded that they never used digital therapy tools, further pointing to the lag in digitalization implementation. Given that students are usually not working clinically, the significant differences observed between them and SLPs is unsurprising. However, the finding that professionals with 1-5 years of experience used digital therapy tools more indicates a growing acceptance of digital eHealth solutions [94]; it could be interesting to investigate in future work what factors specifically contribute to this uptake in younger professionals. Regarding frequency of digital learning tool use, Generation Z (most of whom are students) demonstrated significantly greater digital learning use than Generation Y, which is expected given the shift toward web-based learning during the ongoing COVID-19 pandemic [95].

### Attitudes

Attitudes toward digitalization were primarily positive, with Generation Z demonstrating significantly more positive views than their generational counterparts, as expected per previous research trends [96]. Consistent with previous studies, many respondents indicated uncertainty regarding their preparedness for the digital revolution [15,21,22]. This was especially the case between SLPs and SLP students, which questions whether digital skills are currently learned on the job or through professional development courses; this feeling of unpreparedness among SLP students once again emphasizes the urgent need to incorporate digital skills into clinical curricula, a sentiment also supported by most survey respondents.

Most respondents indicated being open to using digital therapy tools. Interestingly, Generation Z and respondents who were still studying reported being more likely to use digital tools with medical certification than their Generation X counterparts and those with ≥20 years of professional experience. To the best of our knowledge, this specific finding has not been previously reported in the literature. A study with French university students found that students were more comfortable with digital health interventions provided these would be promoted by institutional or official entities, though these opinions were not compared against those of seasoned professionals or older generations [74]. As Generation Z students have been reported to generally demonstrate greater levels of anxiety and need for guidance than previous generations of students, it could be that medical certification through an official entity provides a sense of security and guidance [97]. These findings nevertheless warrant follow-up investigation into the reasons for such perceptions regarding medical certification for digital tools, which may even extend beyond these observed differences in generation and years of experience. Although most respondents agreed that they would pay a reasonable price for a digital therapy app with good ratings, it would be useful to further investigate what respondents define as reasonable, as this loaded question requires deeper investigation.

Intriguingly, a little under one-third of respondents demonstrated skepticism regarding whether such tools provided more advantages than disadvantages and indicated doubts regarding their quality and validity, and a little over half of respondents reported concerns with technical difficulties. These concerns urge for focused research on efficacy and barriers to digital technology use. Current and future physicians and clinicians should be increasingly engaged in systematically evaluating eHealth technologies through tools such as the Mobile App Rating Scale (MARS) [98]. In the German-speaking context, tools such as HealthOn, APPKRI, and APPQ1.0 have emerged for the evaluation of health apps, and within the field of speech-language pathology, the Bewertungskatalog für Apps in Sprachtherapie und Sprachförderung instrument for evaluation of speech-language pathology apps is being optimized and could serve as a future useful evaluative tool [99]. In the optional *other* free response category, doubts regarding patient acceptance, secure data transfer and storage, and ethical consequences were mentioned concerns, which require co-operation with policy and governmental regulation for effective implementation [2,74,75,100,101]. Given reports that 2 in 3 Germans welcome eHealth technologies such as electronic health records and given the increasing number of patient satisfaction studies with digital applications, there appears to be growing patient demand and acceptance of digitalization [90].

In terms of digital learning, most respondents were open to its use, agreed that it offered more advantages than disadvantages, and felt mostly confident with their knowledge of digital learning tools. Interestingly, when reporting concerns, however, 45.3% (77/170) of respondents still questioned the quality of digital learning material, despite previous indications of not being doubtful of quality. This disconnect reveals hesitations in digital uptake and aligns with the reported need to find effective means of verifying the quality and validity of digital learning tools. Although technical standards exist through organizations such as the International Organization for Standardization (ISO) or the Institute of Electrical and Electronics Engineers, these are not immediately comprehensible for clinical instructors, and efforts are still being made to devise frameworks that fit within clinical professional responsibilities and standards [102-104].

### Preferences

In the following study, no significant differences in preferred levels of virtuality, interactivity, and flexibility in terms of time and space were observed across profession, generation, or years of experience. The absence of generational differences aligns with several previous reports [105-107]. Most respondents preferred a blended learning model (half in-class and half virtual) and a limited or moderate to complex level of interactivity. There were almost similar proportions of respondents preferring flexibility in terms of space only (eg, synchronous lectures delivered on the web) or traditional in-person lectures. In a study by Küsel et al [108], German students similarly preferred synchronous web-based learning for directly asking questions; in contrast US students also valued asynchronous options. The German preference toward more traditional means of learning may reflect their general lower reported confidence and perceived readiness for engaging web-based in comparison to their US counterparts [108]. Such differences can be traced to cultural differences in teaching and learning; for example, the US system is known to incorporate more interactive means of learning and virtual learning much earlier on in education [109]. Such findings warrant a deeper investigation into cultural, educational, and societal differences that inevitably affect digitalization uptake, integration, and development in a country. However, it could also be interesting to consider whether the ongoing COVID-19 pandemic contributed to these preferences for more in-person learning because of the potential effects of social isolation and screen fatigue [110]. As Wiederhold [111] identified, factors such as asynchrony of communication even by milliseconds on video calls, lack of nonverbal cues, and additional components (eg, chat function) can introduce additional cognitive load, which can be mentally taxing and result in fatigue. As Bennett et al [112] suggest, having the option to turn off the microphone and fostering environments of inclusiveness and belonging helped to reduce this fatigue and are crucial elements to include moving forward in an uncertain digital future.

### Limitations

Critically, this study must be interpreted in light of its limitations. First, this cross-sectional investigation was conducted in the German-speaking population and thus may have limited external validity to other cultural and geographical contexts. However, as digitalization is a global phenomenon, we hope that our findings shed some light on factors that could be playing into common trends, areas of opportunity, and barriers to the uptake of digital technologies. It is also important to note that the stark contrast in the number of female versus male participants can likely be attributed to the fact that the field of SLP is predominantly female (93%) and the number of female otorhinolaryngologists has been steadily rising (35.7% as of 2020) in Germany [113,114]. In addition, this was an open-ended survey that consisted of a convenience sample, which could have attracted individuals who were already more inclined toward taking interest in digitalization. Given this convenience sample, it was difficult to control for equal group sizes, though statistical adjustments were made, and the sample size of the study is relatively small. Our aim was primarily to obtain a comprehensive overview of the current state of knowledge, use, attitudes, and preferences regarding digital therapy and learning in CSD as such data were not previously available; thus, the survey data have only begun to scratch the surface of current trends, and furthermore deep investigation into underlying factors contributing to digital experiences, uptake, and progress is crucial. Given the rapid rate of digital progress, this survey study also offers just a snapshot of the state of digitalization and stakeholder opinions at the time of study.

### Future Directions

As a survey study, the following investigation analyzed perceptions regarding digital therapy and learning, though it would be important in future studies to also measure actual knowledge, implementation, and use. In moving forward toward an uncertain digital future in which the evidence base for new technologies has yet to be developed or may even be rendered obsolete before one can even be formed, it will be essential to engage in *adaptive* education and practice. An incorporation of digital skills into clinical curricula will be necessary as the clinical landscape continues to evolve. It has been previously recommended, for example, that students begin to also receive formal coursework in data analytics, governance, and privacy as well as emerging models of care such as remote monitoring, self-management, and increasing telepractice [67]. Data visualizations could be used for problem-based learning, digital applications could be incorporated into treatment planning as part of collaborative clinical research, and current and future professionals must inevitably take part in patient empowerment and education through digital means [12,21,67]. To begin this process, however, clinical instructors must also receive systematic training in these areas, and more data must be collected regarding current gaps in access and professional and student needs. Further research into effective implementation could be informed through increasing interdisciplinary strategizing within and outside of CSD [115]. As collegial and organizational support has been previously shown to be essential for creating positive experiences with technology, it will be critical that practical supports for innovation are carefully planned and empirically substantiated to the best extent possible [2].

Our study has demonstrated that students and professionals in CSD continue to feel uncertain regarding their knowledge of digital health and digital learning, are still using more traditional devices, software or systems, and formats, though are interested in exploring more interactive, digital means for teaching, learning, and practice. They have understandable concerns surrounding quality and validity of digital resources, data privacy, technical complexity, social engagement, and effective implementation. These concerns amidst the rapidly changing digital landscape urges for the expedited exploration of empirically substantiated, though flexible, solutions.

## Appendix

Multimedia Appendix 1 Survey screens (in German).

## Abbreviations

AAC: augmentative and alternative communication
CSD: communication sciences and disorders
SLP: speech-language pathologist
VR: virtual reality
