# Supplementary material for: Digital Health and Digital Learning Experiences Across Speech-Language Pathology, Phoniatrics, and Otolaryngology: Interdisciplinary Survey Study
Source: JMIR Med Educ. 2021 Nov 5;7(4):e30873. doi: 10.2196/30873 (PMC8663699; doi:10.2196/30873)
Supplement: Multimedia Appendix 1 [file mededu_v7i4e30873_app1.pdf]

Multimedia Appendix 1. Survey Screens (*in German*)

TEIL I

Soziodemografische Informationen

*In welchem Beruf arbeiten Sie zurzeit?

Bitte wählen Sie eine der folgenden Antworten:

- Arzt/Ärztin (Phoniatrie/HNO)
- Logopäde/Logopädin oder akademische(r) Sprachtherapeut/Sprachtherapeutin
- Studierende der Logopädie/Sprachtherapie
- Studierende der Humanmedizin
- Sonstiges:

*Wie lange arbeiten Sie bereits in diesem Beruf?

Bitte wählen Sie eine der folgenden Antworten:

- Ich studiere noch
- 1-5 Jahre
- 6-10 Jahre
- 11-15 Jahre
- 16-20 Jahre
- Mehr als 20 Jahre

*Welcher Generation gehören Sie an?

Bitte wählen Sie eine der folgenden Antworten:

- Generation Z (Geburtsjahre ab 1996)
- Generation Y Millennials (Geburtsjahre 1980 - 1995)
- Generation X (Geburtsjahre 1965 - 1979)
- Babyboomer (Geburtsjahre 1946 - 1964)

*Geschlecht:

Bitte wählen Sie eine der folgenden Antworten:

- männlich
- weiblich
- divers

Zurück

Weiter

ERSTER TEIL: Vorkenntnisse und Einsatz 1

*Kennen Sie die Begriffe "Digital Health" oder "E-Health"?

Bitte wählen Sie eine der folgenden Antworten:

- Nein, ich habe noch nie von diesen Begriffen gehört
- Ja, ich habe von ihnen gehört, bin mir aber nicht sicher, was sie bedeuten
- Ja, ich habe von ihnen gehört, habe aber nur begrenzte Kenntnisse
- Ja, und ich bin überzeugt, dass ich den Bereich Digital Health / E-Health gut kenne

*Kennen Sie die Begriffe „Digital Learning“ oder „E-Learning“?

Bitte wählen Sie eine der folgenden Antworten:

- Nein, ich habe noch nie von diesen Begriffen gehört
- Ja, ich habe von ihnen gehört, bin mir aber nicht sicher, was sie bedeuten
- Ja, ich habe von ihnen gehört, habe aber nur begrenzte Kenntnisse
- Ja, und ich bin überzeugt, dass ich den Bereich Digital Learning/ E-Learning gut kenne

Zurück Weiter

Vorkenntnisse und Einsatz 2: Geräte

Für den Zweck dieser Umfrage kann unter „Digital Health / E-Health" die Verwendung von Technologie oder digitalen Medien zur Förderung, Aufrechterhaltung oder Steuerung der Gesundheit einer Person definiert werden.

Für den Zweck dieser Umfrage werden unter „Digital Learning / Digitales Lernen / E-Learning" alle Lernformen definiert, bei denen elektronische oder digitale Medien zur Unterstützung von Lernprozessen verwendet werden, in diesem Fall insbesondere im medizinischen oder klinischen Bildungskontext.

*Welche Geräte verwenden Sie regelmäßig für berufliche Zwecke oder für Ihr Studium?

Mehrfachnennung möglich

Bitte wählen Sie die zutreffenden Antworten aus:

- [ ] Computer
- [ ] Smartphone
- [ ] MP3 Player (z.B. iPod)
- [ ] Tablet (Apple oder Android)
- [ ] E-Reader
- [ ] (tragbare) Spielkonsole (z.B. Xbox, PS3, Nintendo Wii oder Switch, PSP)
- [ ] Virtual Reality Systeme (z.B. Oculus, HTC, Playstation VR)
- [ ] Sonstiges: ______

Zurück

Weiter

Vorkenntnisse und Einsatz 3: Software

*Welche der folgenden studium- oder berufsbezogenen Software verwenden Sie regelmäßig?

Mehrfachnennung möglich

Bitte wählen Sie die zutreffenden Antworten aus:

- [ ] Textverarbeitungssoftware (z.B. Microsoft Word)
- [ ] Präsentationssoftware (z.B. Microsoft PowerPoint)
- [ ] Tabellenkalkulationssoftware (z.B. Microsoft Excel)
- [ ] Statistik- und Analyse-Software (z.B. SPSS, SAS, R)
- [ ] Datenbank (z.B. Scopus, PubMed, Medline, Springer)
- [ ] Videokonferenz-Plattform (z.B. Skype, Zoom, Google Hangouts/Meet, Microsoft Teams)
- [ ] Elektronische Patientenakte
- [ ] E-Rezept / Heilmittelverordnung
- [ ] Online-Terminplaner und/oder Online-Terminbuchung
- [ ] Online Sprechstunde / Telekonsile (Telemedizin)
- [ ] Medizinische Abrechnungssoftware
- [ ] Krankenhausinformationssystem (z.B. Medico)
- [ ] Sicheres medizinisches Kommunikationssystem
- [ ] Sonstiges: ______

Zurück

Weiter

Vorkenntnisse und Einsatz 4: Formate

*Welche digitalen Formate haben Sie bereits zum Lernen/Lehren und/oder zuvor in der klinischen Praxis verwendet?

Mehrfachnennung möglich

Bitte wählen Sie die zutreffenden Antworten aus:

- [ ] Podcasts
- [ ] Videos
- [ ] E-Books
- [ ] Apps
- [ ] 3D Modelle
- [ ] Webseiten
- [ ] Soziale Netzwerke
- [ ] Online Seminare oder Kurse
- [ ] Serious Games / Game-Based Learning
- [ ] Simulationen
- [ ] 3D Welten (Virtual Reality)
- [ ] Sonstiges: ______

*Mit welchen E-Learning-Formaten könnten Sie sich vorstellen, ***zukünftig*** zu lernen, zu lehren oder sie in Ihre Praxis zu integrieren?

Mehrfachnennung möglich

Bitte wählen Sie die zutreffenden Antworten aus:

- [ ] Podcasts
- [ ] Videos
- [ ] E-Books
- [ ] Apps
- [ ] 3D Modelle
- [ ] Webseiten
- [ ] Soziale Netzwerke
- [ ] Online Seminare oder Kurse
- [ ] Serious Games / Game-Based Learning
- [ ] Simulationen
- [ ] 3D Welten (Virtual Reality)
- [ ] Sonstiges: ______

Zurück   Weiter

Vertrautheit und Verwendung 1: E-Learning Tools

Die folgenden Fragen beziehen sich auf Ihre Vertrautheit mit und Ihre Verwendung von „E-Learning-Tools".

Für den Zweck dieser Umfrage werden E-Learning-Tools als elektronische oder digitale Medien (z. B. Website, App, Podcast, Online-Kurse, Lernmanagementsystem) definiert, die für Lernzwecke verwendet werden können - für die Ausbildung und / oder für die Fort- oder Weiterbildung.

*Kennen Sie bereits ***E-Learning Tools*** für das Studium, die Lehre oder Fortbildungen?

Ja | Nein

Welche E-Learning Tools kennen Sie schon? Wie fanden Sie diese Tools?

*Wie oft nutzen Sie digitale ***Lernangebote/E-Learning Tools*** für Ihr Lernen oder Ihre Fort-/Weiterbildung?

Bitte wählen Sie eine der folgenden Antworten:

- nie
- monatlich
- wöchentlich
- täglich

Zurück | Weiter

Vertrautheit und Verwendung 1: Digitale Therapie Tools

Die folgenden Fragen beziehen sich auf Ihre Vertrautheit mit und Ihre Verwendung von „digitalen Therapie-Tools".

Für den Zweck dieser Umfrage werden digitale Therapie-Tools als elektronische oder digitale Medien definiert, die für klinische Zwecke oder klinische Forschungszwecke (z. B. Gesundheitsüberwachung, Diagnose, Therapieübungen) verwendet werden können. Häufige Beispiele sind Smartwatches, Fitness-Apps und Therapie-Apps.

*Kennen Sie bereits ***digitale Therapie-Tools*** (z.B. Apps, Online-Websites, Simulationen)?

Ja | Nein

Welche digitalen ***Therapie-Tools*** kennen Sie bereits? Wie fanden Sie diese Tools?

*Wie oft setzen Sie ***digitale Therapie-Tools*** in der klinischen Praxis ein?

Bitte wählen Sie eine der folgenden Antworten:

- nie
- monatlich
- wöchentlich
- täglich
- Ich arbeite derzeit nicht klinisch

Zurück | Weiter

Vertrautheit und Verwendung 2: E-Learning Tools, Einstellungen

*Bitte bewerten Sie die folgenden Angaben in Bezug auf ***E-Learning Tools***

	stimme nicht zu	stimme eher nicht zu	stimme eher zu	stimme zu
Ich bin offen für den Einsatz von E-Learning Tools	○	○	○	○
Ich glaube, E-Learning bietet mehr Vor- als Nachteile und hat das Potenzial, das Lernen zu individualisieren	○	○	○	○
Ich bezweifle die Qualität und Zuverlässigkeit von E-Learning-Tools	○	○	○	○
Ich würde E-Learning-Angebote eher nutzen, wenn sie von einer akademischen Einrichtung oder einem Berufs- und Fachverband zertifiziert oder angeboten würden.	○	○	○	○
Ich bin zuversichtlich hinsichtlich meiner Kenntnisse über digitale E-Learning Tools und Angebote	○	○	○	○

*Ich habe die folgenden Bedenken in Bezug auf E-Learning-Tools, falls vorhanden (Mehrfachnennung möglich)

Bitte wählen Sie die zutreffenden Antworten aus:

- ☐ keine Bedenken
- ☐ technische Schwierigkeiten
- ☐ Mangelnde Qualität des Lernmaterials
- ☐ Schwierigkeiten mit Selbstdisziplin oder Lernkompetenz
- ☐ Bedenken hinsichtlich potenziell reduzierter sozialer Interaktionen
- ☐ Sonstiges: ________

Zurück | Weiter

Vertrautheit und Verwendung 3: Präferenzen

*Was wäre Ihre ideale Lernumgebung? (Grad der Virtualität)

Bitte wählen Sie eine der folgenden Antworten:

- **Präsenzlernen**: das Lernen findet ausschließlich persönlich in einem physischen Hörsaal/Klassenzimmer statt
- **Präsenz begleitend**: das Lernen findet hauptsächlich im Hörsaal oder Klassenzimmer statt, und gelegentlich werden zusätzliche elektronische Materialien zur Verfügung gestellt
- **Blended Learning**: eine Kombination aus Online-Lernen und Präsenzlernen, bei der beide Methoden komplementär eingesetzt werden, um ein umfassendes Lernerlebnis zu schaffen
- **Inverted Classroom**: die üblichen Aktivitäten innerhalb und außerhalb des Hörsaals/Klassenzimmers werden „umgedreht". Das Lernen findet hauptsächlich virtuell durch online zur Verfügung gestellte Inhalte/Lernmaterial. Ziel der Präsenzveranstaltung ist es, das Erlernte zu vertiefen und neues Wissen anzuwenden.
- **Virtuelles Lernangebot (Online Seminar/Kurs)**: das Lernen findet ausschließlich virtuell statt

*Welchen Grad an Interaktivität bevorzugen Sie am meisten?

Bitte wählen Sie eine der folgenden Antworten:

- **Passiv**: Website mit Text und statischen Grafiken, Podcasts, E-Books, Videos
- **Begrenzt**: Websites mit anklickbaren Medien, Drag-and-Drop-Interaktionen, Animationen
- **Moderat/Komplex**: Online-Kurse mit anpassbaren Tools und szenariobasierten Fällen mit mehreren Bildschirmen, manipulierbaren Objekten (3D-Modelle)
- **Fortgeschritten/Aktiv**: Szenariobasierte VR-Simulationsübungen mit 3D-Manipulationsfunktionen, Game-Based Learning mit Avataren, Echtzeit-Fortschrittsanzeigen

*In Bezug auf räumliche und zeitliche Flexibilität, was ist Ihre bevorzugte Lernmethode?

__Synchron__ bezieht sich auf Lernen, das in Echtzeit stattfindet. ***Asynchron*** bedeutet, dass das Lernen zeitunabhängig stattfinden kann

Bitte wählen Sie eine der folgenden Antworten:

- **zeit-und ortsabhängig**: Veranstaltung oder Vorlesung im Hörsaal / Seminarraum
- **zeitunabhängig**: Simulationslabor, Computerraum
- **ortsunabhängig**: synchrone Online-Veranstaltungen (Kurse/Webinare/Seminare), Vorlesungsaufzeichnungen, Game-Based Learning, Virtual Reality (VR), 3D Welten
- **zeit-und ortsunabhängig**: asynchrone Online-Veranstaltungen (Kurse/Webinare/Seminare), e-Books, Apps, Podcasts, Virtual Reality (VR)

Zurück | Weiter

Vertrautheit und Verwendung 4: Digitale Therapie Tools, Einstellungen

*Bitte bewerten Sie die folgenden Angaben in Bezug auf ***digitale Therapie-Tools***

	stimme nicht zu	stimme eher nicht zu	stimmer eher zu	stimme zu
Ich bin offen für den Einsatz von digitale Therapie-Tools	○	○	○	○
Ich glaube, dass digitale Therapie Tools mehr Vor- als Nachteile bieten und das Potenzial haben, die Therapie zu individualisieren	○	○	○	○
Ich bezweifle die Qualität oder Zuverlässigkeit digitaler Therapie Tools	○	○	○	○
Ich würde eher digitale Therapie-Tools verwenden, wenn sie medizinisch zertifiziert wären	○	○	○	○
Ich wäre bereit, einen angemessenen Preis für ein digitales Therapie-Tool zu zahlen, wenn es gute Bewertungen hätte	○	○	○	○

*Ich habe die folgenden Bedenken hinsichtlich ***digitaler Therapie-Tools***, falls vorhanden (Mehrfachnennung möglich)

Bitte wählen Sie die zutreffenden Antworten aus:

- ☐ Keine Bedenken
- ☐ Bedenken hinsichtlich technischer Schwierigkeiten
- ☐ Bedenken hinsichtlicht der Validität des Tools
- ☐ Bedenken hinsichtlich der diagnostischen oder therapeutischen Qualität
- ☐ Sonstiges: ________

Zurück

Weiter

Gesamteindruck

*Bitte bewerten Sie die folgenden Angaben:

	stimme nicht zu	stimme eher nicht zu	stimme eher zu	stimme zu
Generell sehe ich die Digitalisierung in der Medizin positiv	◯	◯	◯	◯
Ich fühle mich im Allgemeinen gut vorbereitet auf die digitale Revolution im Lernen und in der klinischen Praxis	◯	◯	◯	◯
Die Themen E-Health, digitale Tools und Fähigkeiten sollen in Zukunft stärker in unsere Aus-, Fort- und Weiterbildung einbezogen werden	◯	◯	◯	◯

Zurück Weiter

Gesamteindruck 2

Haben Sie weitere Vorschläge oder Kommentare zur Umfrage?

Zurück Absenden
